# Molecular Pharmacology of P2X Receptors: Exploring Druggable Domains Revealed by Structural Biology

**DOI:** 10.3389/fphar.2022.925880

**Published:** 2022-06-17

**Authors:** Adam C. Oken, Ipsita Krishnamurthy, Jonathan C. Savage, Nicolas E. Lisi, Michael H. Godsey, Steven E. Mansoor

**Affiliations:** ^1^ Department of Chemical Physiology and Biochemistry, Oregon Health & Science University, Portland, OR, United States; ^2^ Knight Cardiovascular Institute, Oregon Health & Science University, Portland, OR, United States

**Keywords:** purinergic receptors, P2X, ion channels, cryo-EM, X-ray crystallography, antagonist, allosteric modulator, structure-based drug design

## Abstract

Extracellular ATP is a critical signaling molecule that is found in a wide range of concentrations across cellular environments. The family of nonselective cation channels that sense extracellular ATP, termed P2X receptors (P2XRs), is composed of seven subtypes (P2X_1_-P2X_7_) that assemble as functional homotrimeric and heterotrimeric ion channels. Each P2XR is activated by a distinct concentration of extracellular ATP, spanning from high nanomolar to low millimolar. P2XRs are implicated in a variety of physiological and pathophysiological processes in the cardiovascular, immune, and central nervous systems, corresponding to the spatiotemporal expression, regulation, and activation of each subtype. The therapeutic potential of P2XRs is an emerging area of research in which structural biology has seemingly exceeded medicinal chemistry, as there are several published P2XR structures but currently no FDA-approved drugs targeting these ion channels. Cryogenic electron microscopy is ideally suited to facilitate structure-based drug design for P2XRs by revealing and characterizing novel ligand-binding sites. This review covers structural elements in P2XRs including the extracellular orthosteric ATP-binding site, extracellular allosteric modulator sites, channel pore, and cytoplasmic substructures, with an emphasis on potential therapeutic ligand development.

## Introduction

Membrane proteins that recognize extracellular purine nucleotides, termed purinergic receptors, are grouped into three unique families: G-protein coupled P1 receptors that recognize adenosine, G-protein coupled P2Y receptors that recognize ADP and ATP, and ligand-gated P2X receptor (P2XR) ion channels that recognize ATP exclusively (Burnstock, 1976; van Calker et al., 1979; Londos et al., 1980; Webb et al., 1993; Brake et al., 1994; Valera et al., 1994). The seven P2XR subtypes, denoted P2X_1_-P2X_7_, are trimeric non-selective cation channels that are activated by distinct extracellular concentrations of ATP, a key signaling molecule released from cells in a broad range of physiological and pathophysiological states, from low concentrations during homeostasis to high concentrations in chronic inflammation or ischemia (Burnstock, 1972; Roman and Fitz, 1999; Taylor et al., 1999; Burnstock, 2004). Individual P2XR subtypes are activated by a wide range of distinct extracellular ATP (eATP) concentrations, from high nanomolar to low millimolar (Jarvis and Khakh, 2009; Illes et al., 2021). The binding of eATP to the receptor induces a conformational change, opening the ion channel and facilitating the influx of Na^+^ and Ca^2+^ ions and the efflux of K^+^ ions (Samways et al., 2014). The resultant net inward current following P2XR activation plays a significant role in downstream signaling and cellular function.

The functional and clinical significance of P2XR subtypes is related to both their individualized affinity to eATP and the cell types in which they are expressed. These include, but are not limited to, platelets (P2X_1_), smooth muscle cells (P2X_1-7_, predominantly P2X_1_), sensory neurons (P2X_1-7_, predominantly P2X_3_), epithelial cells (P2X_4_, P2X_5_, P2X_6_, P2X_7_), and immune cells (P2X_4_ and P2X_7_) (Burnstock and Kennedy, 2011). Accordingly, P2XRs are implicated in an array of pathological conditions. For instance, in animal models of Alzheimer’s disease (AD), pharmacologic inhibition or genetic depletion of P2X_7_ significantly improves the symptoms and neuropathology of AD (Francistiova et al., 2020). Several other examples of P2XR-implicated pathological conditions include: platelet aggregation (P2X_1_), hearing loss (P2X_2_), asthma (P2X_3_), vascular inflammation (P2X_7_), and cancer (P2X_7_) (Kamei et al., 2005; Baroni et al., 2007; Burnstock and Kennedy, 2011; Furlan-Freguia et al., 2011; Mahaut-Smith et al., 2011; North and Jarvis, 2013; Yan et al., 2013; Burnstock and Knight, 2018; Lara et al., 2020; Illes et al., 2021). As a result, P2XRs are an active area of therapeutic research with several subtype-specific antagonists currently in clinical trials to treat persistent cough (clinicaltrials.gov: NCT02502097), rheumatoid arthritis (clinicaltrials.gov: NCT00628095), and depression (clinicaltrials.gov: NCT04116606), among others (Kamei et al., 2005; Pfizer, 2008; Keystone et al., 2012; Abdulqawi et al., 2015; Afferent Pharmaceuticals, 2015; CCTU-Core et al., 2019; Richards et al., 2019).

P2XRs assemble as both homotrimeric and heterotrimeric channels. The known heterotrimeric assemblies include: P2X_1/2_, P2X_1/4_, P2X_1/5_, P2X_2/3_, P2X_2/6_, P2X_4/5_, P2X_4/6_, and P2X_4/7_ (Murrell-Lagnado and Qureshi, 2008). Very little is known about heterotrimeric composition, stoichiometry, and function. This is compounded by the increased pharmacologic complexity of heterotrimeric receptors, which poses an additional challenge to therapeutic targeting of P2XR activity. For example, heterotrimeric P2X_2/3_ has a ligand dose-response profile that is distinct from either homotrimeric P2X_2_ or P2X_3_ (Lewis et al., 1995). Currently, there are published structures for only three of seven homotrimeric subtypes and none for heterotrimeric receptors. The first P2XR structure, solved by X-ray crystallography, characterized the apo (unbound) closed state, defined the overall architecture of this receptor family, and confirmed three-fold symmetry for the homotrimeric assembly (Kawate et al., 2009). Subsequent crystallographic studies have identified the extracellular orthosteric ATP-binding site, revealed two distinct extracellular allosteric binding sites, and defined the molecular mechanisms of P2XR gating (Hattori and Gouaux, 2012; Karasawa and Kawate, 2016; Mansoor et al., 2016; Kasuya et al., 2017; Wang et al., 2018). While these crystallographic studies established a foundational understanding of P2XR structure and function, truncation of the cytoplasmic N- and C- termini (necessary for crystallization) limited the scope of our understanding of P2XR biology. The application of single-particle cryogenic electron microscopy (cryo-EM) to rat P2X_7_ overcame these limitations and provided visualization of the first full-length, wild-type P2XR, revealing novel features in the cytoplasmic domain that are essential for receptor function (McCarthy et al., 2019).

The “resolution revolution” in cryo-EM is advancing the ability of researchers to solve high-resolution protein structures (Kuhlbrandt, 2014). Compared to X-ray crystallography, cryo-EM requires less protein, tolerates more sample heterogeneity, and allows for the study of proteins in more native-like membrane environments, all of which are challenges in studying membrane protein structure (Su et al., 2021). Moreover, cryo-EM structures of wild-type proteins and protein complexes are consistently reaching sufficient resolutions to discover novel ligands and identify post-translational modifications (McCarthy et al., 2019; Flores et al., 2020; Nakane et al., 2020; Rahman et al., 2020). In turn, these new high-resolution structures of therapeutically relevant proteins have accelerated structure-based drug design (SBDD) efforts, which are significantly more efficient and generate higher-specificity ligands when compared to classical *in vitro* based assays (Lionta et al., 2014; Batool et al., 2019; Ballante et al., 2021; Ford et al., 2021; Lees et al., 2021). Cryo-EM is well suited to visualize novel receptor structures and receptor-ligand interactions for use in SBDD techniques, ultimately leading to more potent therapeutic compounds with fewer and less severe side effects for patients.

Careful examination of the available P2XR structures provides insights into the molecular pharmacology of this therapeutically relevant receptor family. This review highlights both known and potentially targetable sites within P2XRs that are critical to modulate receptor function.

### OVERALL P2XR DOMAIN ARCHITECTURE

**FIGURE 1:**
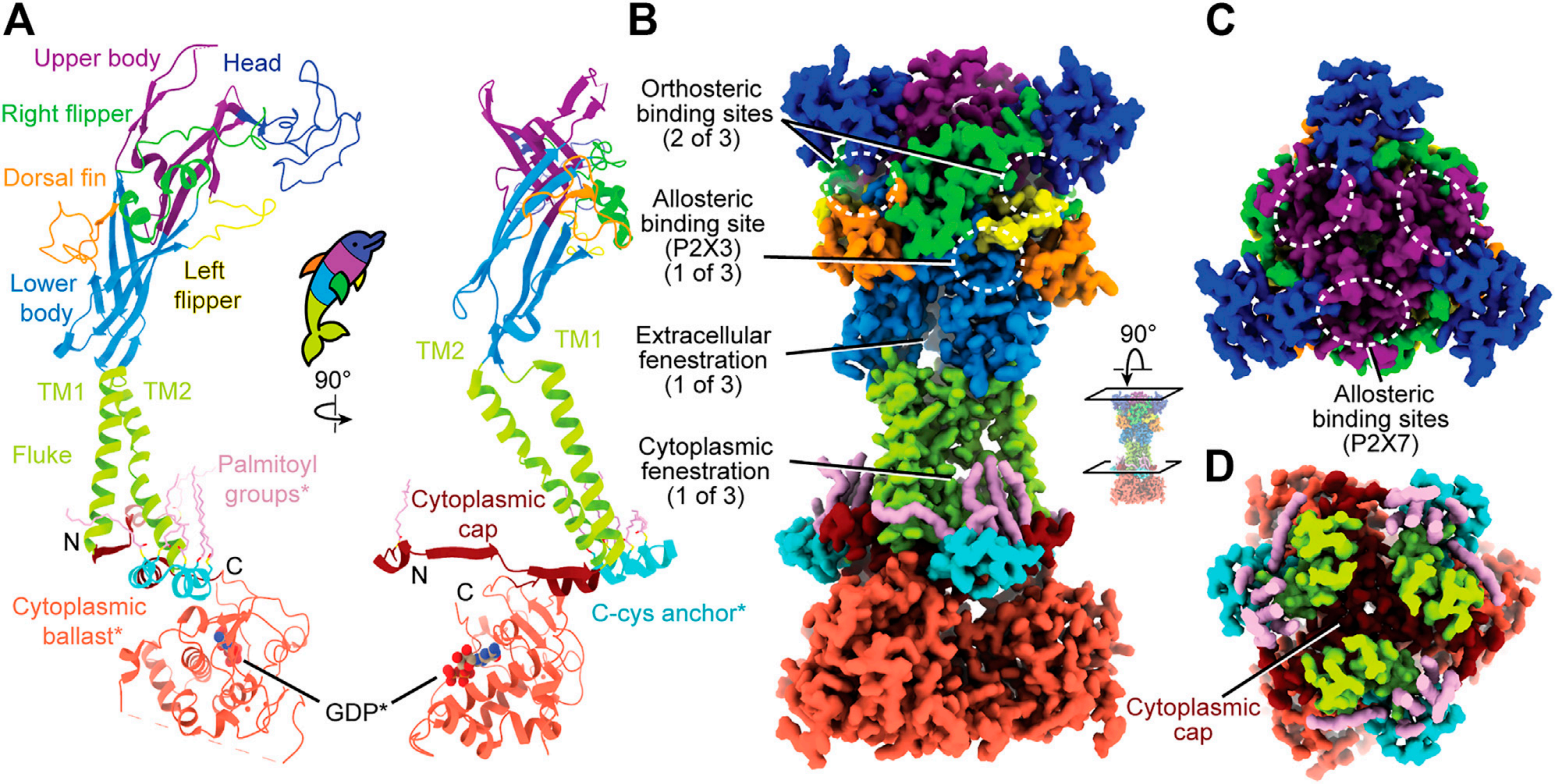
Overall, dolphin-resembling architecture of P2XRs exemplified by rP2X_7_ (PDB: 6U9V). **(A)** *Left:* Ribbon representation of one subunit of rP2X_7_ colored by domain architecture. *Right:* Orthogonal view colored by domain architecture. **(B)** Surface representation of the biological trimeric assembly of rP2X_7_ colored by domain architecture, viewed parallel to the membrane and with select structural elements labeled. The orthosteric ATP-binding site and the extracellular and cytoplasmic fenestrations are structurally conserved amongst P2XRs. The allosteric site found in hP2X_3_ is mapped to its respective spatial position on the presented P2X_7_ model. This allosteric site has not been confirmed to exist in P2X_7_, but the structural similarity amongst P2XRs allows visualization of its relative position. **(C)** Top-down view of the trimeric assembly of the rP2X_7_ extracellular domain colored by domain architecture, with denoted symmetry-related allosteric site confirmed in P2X_7_. This view highlights the three identical, symmetry-related, allosteric binding sites found on top of the extracellular domain of homotrimeric P2X_7_. **(D)** Biological trimeric assembly of rP2X_7_, colored by domain architecture, viewed perpendicular to the membrane from the center of the transmembrane domain into the cytoplasm. *Denotes structural elements found only in the P2X_7_ subtype.

The first P2XR structure to be solved, the apo closed state of a truncated zebrafish P2X_4_ (zfP2X_4_), defined the architecture of the extracellular and transmembrane regions for this receptor family (Kawate et al., 2009). Within each protomer of a trimeric receptor, the extracellular and transmembrane domains were imagined to resemble a breaching dolphin (Figure 1A) (Kawate et al., 2009). This overall architecture was confirmed to be conserved for apo closed states of the human P2X_3_ (hP2X_3_), giant panda P2X_7_ (pdP2X_7_), and rat P2X_7_ (rP2X_7_) receptors in successive structural studies (Karasawa and Kawate, 2016; Mansoor et al., 2016; McCarthy et al., 2019). Comparing the molecular architecture of several P2XRs reveals conserved sites that are targetable across this receptor family and highlights the structural differences across subtypes.

#### The Extracellular Domain

The extracellular domain, which has been likened to a dolphin’s head and body, is composed of thirteen β-strands and four α-helices (Figure 1A). The β-sheets of the lower and upper body compose the central backbone of the extracellular domain of all P2XRs, with peripheral α-helices and loops variable between subtypes. Tucked within the loops, β-sheets, and α-helices of the extracellular domain are several ligand-binding pockets that modulate P2XR function. The orthosteric ATP-binding site, defined as the location of endogenous ligand (eATP) binding, has been structurally characterized for three P2XR subtypes: hP2X_3_, zfP2X_4_, and rP2X_7_ (Mansoor et al., 2016; Kawate et al., 2009; Hattori and Gouaux, 2012; McCarthy et al., 2019). This site is found in all P2XRs at an interface formed by the head, left flipper, and upper body of one protomer and the lower body of the neighboring protomer (Figures 1A,B). Thus, a functional trimeric P2XR has three symmetry-related orthosteric binding sites. However, it is generally accepted that the occupation of all three of these binding sites by ATP is not necessary to activate the channel (Friel and Bean, 1988; Coddou et al., 2011; Kawate, 2017).

In addition, extracellular allosteric binding sites—distinct from the orthosteric binding site—have been structurally confirmed in hP2X_3_ and pdP2X_7_ and are predicted in other P2XRs (Figures 1B,C) (Ase et al., 2019; Bidula et al., 2022; Karasawa and Kawate, 2016; Obrecht et al., 2019; Wang et al., 2018). One structurally confirmed allosteric site in hP2X_3_ is found towards the base of the extracellular domain, formed by the left flipper of one subunit and the lower body and dorsal fin of another (Figure 1B) (Wang et al., 2018). Due to symmetry, there are three such allosteric sites in homotrimeric hP2X_3_ (Figure 1B). Another allosteric site, structurally confirmed in pdP2X_7_, is located on top of the extracellular domain and is formed by the upper body between neighboring protomers (Figure 1C) (Karasawa and Kawate, 2016). Similarly, due to symmetry, there are three such allosteric sites in homotrimeric pdP2X_7_ (Figure 1C). Ligand binding at allosteric sites modulates protein activity through structural rearrangements, resulting in either inhibited (negative allostery) or enhanced (positive allostery) orthosteric ligand binding. Negative allosteric modulators are non-competitive antagonists as they do not compete with orthosteric ligand binding. The orthosteric and two distinct allosteric sites are critical targets to consider when developing potential therapeutic ligands that modulate P2XR activity.

#### The Transmembrane Domain

The transmembrane (TM) domain, the dolphin’s tail, is composed of two α-helices (the peripheral TM1 and internal, pore-lining TM2) that span the membrane, connecting the extracellular and cytoplasmic domains (Figures 1A,B). Together, the six TM helices of the trimeric receptor form the pore and undergo distinct conformational rearrangements upon ATP binding (Li et al., 2010). The three conformational states of the gating cycle include the apo closed state, the ATP-bound open state, and the ATP-bound desensitized (closed) state (Mansoor et al., 2016). Desensitization refers to progressive pore closure during sustained agonist binding, which has been thoroughly reviewed for P2XRs (Werner et al., 1996; North, 2002; Kawate, 2017; Mansoor, 2022, in press). The rearrangements of the pore during the gating cycle are described in the helical recoil model of receptor desensitization, first proposed from the published structures of hP2X_3_ (Mansoor et al., 2016). The P2X_7_ subtype is the only P2XR that does not undergo desensitization. Currently, there are no structures characterizing P2XR pore-modulating ligands, but such a class of antagonists would have therapeutic potential for modulating P2XR function.

#### The Cytoplasmic Domains

Not much is known about the structure of cytoplasmic domains for the majority of P2XR subtypes. However, the hP2X_3_ ATP-bound open state structure and the full-length rP2X_7_ structures highlight aspects of intracellular elements (Mansoor et al., 2016; McCarthy et al., 2019). The first segment of the cytoplasmic domain was elucidated by the hP2X_3_ ATP-bound open state structure, which revealed the cytoplasmic ends of the TM helices are flanked by a substructure termed the “cytoplasmic cap” (Figures 1A,D) (Mansoor et al., 2016). The cytoplasmic cap is a product of domain swapping, composed of intertwining β-strands from all three protomers knitted together to form β-sheets that run parallel to the membrane. The stability of the cytoplasmic cap plays a pivotal role in the gating cycle of distinct subtypes by setting the rate of desensitization (Mansoor et al., 2016). Two additional cytoplasmic structural features unique to P2X_7_ were revealed by cryo-EM, a cysteine rich domain termed the “C-cys anchor“ and a ∼200 residue C-terminal domain termed the “cytoplasmic ballast” (Figures 1A,B) (McCarthy et al., 2019). Further elucidation of P2XR cytoplasmic structural diversity by cryo-EM will provide invaluable information about receptor biology.

The array of P2XR structures to date have supplemented our understanding of P2XR function and defined orthosteric and allosteric ligand binding, the path of ion flow through the pore, and the helical recoil model of desensitization. The recent discoveries of the previously unknown cytoplasmic elements of rP2X_7_, facilitated by cryo-EM, have raised new questions regarding its metabotropic signaling properties (Surprenant et al., 1996; Wilson et al., 2002; Cheewatrakoolpong et al., 2005; Adinolfi et al., 2010; Costa-Junior et al., 2011; McCarthy et al., 2019). The nuances of the structural elements contained within the P2XR architecture are discussed below, focusing on potential strategies to target them therapeutically with ligands.

## P2XR Sequence Conservation

**FIGURE 2:**
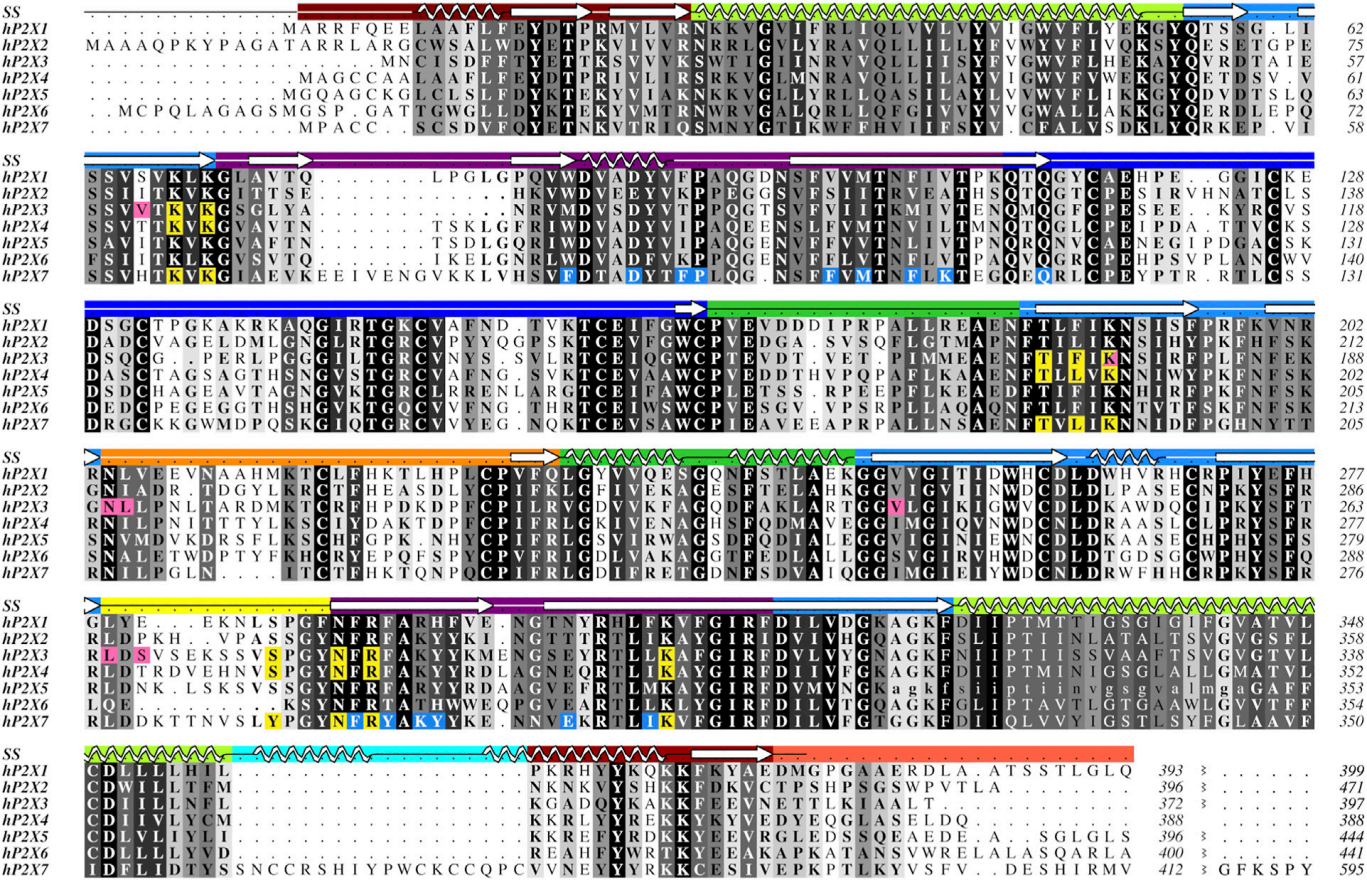
Protein sequence alignment of the seven human P2XR subtypes, P2X_1_-P2X_7_. Amino acids are shown in grayscale by level of sequence conservation across human subtypes as calculated with Alscript (Barton, 1993) (white text on black background denotes full sequence conservation). Residues with direct ligand interactions are highlighted, with the orthosteric ATP-binding site residues in yellow and the residues of the two distinct allosteric sites in pink and blue. These residues are highlighted only for subtypes for which there are published structures. Secondary structure (SS) is denoted in the top row and is colored by domain architecture corresponding to the color scheme in Figure 1. The break at the end of the sequence alignment condenses the divergent cytoplasmic domains. The ancestral difference in TM2 of hP2X_5_, as described in the text, is indicated with lower case letters. The sequence alignment was generated in Clustal Omega and the figure created using Aline (Bond and Schuttelkopf, 2009; Sievers and Higgins, 2018). Sequences were obtained from the UniProt database with accession numbers P51575, Q9UBL9, P56373, Q99571, Q93086, O15547, and Q99572 for hP2X_1_-hP2X_7_, respectively.

Protein sequence alignments of human P2XRs reveal these receptors share ∼35–53% sequence identity between any two subtypes (Figure 2). P2XRs vary in length by up to 207 amino acids per protomer, with P2X_7_ having the longest sequence and P2X_4_ having the shortest (595 and 388 amino acids per protomer, respectively). The most significant variations between P2XR sequences lay in their cytoplasmic termini, which are known to play a critical role in receptor desensitization, trafficking, and signaling (Brandle et al., 1997; Koshimizu et al., 1999; Boue-Grabot et al., 2000; North, 2002; Chaumont et al., 2004; Jarvis and Khakh, 2009; Hausmann et al., 2014; Mansoor et al., 2016; Sattler and Benndorf, 2022). The N-termini vary in length by up to 18 amino acids, with P2X_2_ having the longest N-terminus at 20 residues before the first α-helix of the cytoplasmic cap and P2X_3_ having the shortest at only two residues. The C-termini vary in length by up to 191 amino acids after the last β-strand in the cytoplasmic cap, with P2X_7_ having the longest C-terminus at 203 residues and P2X_4_ having the shortest at 12 residues. Despite significant sequence similarity between P2XRs, subtype-specific sequence differences confer distinct receptor functionality. As our interests lie in human specific drug design, unless stated otherwise, all residues discussed will be from the register of human orthologs.

There is no published structure of P2X_5_ but this subtype is known to be expressed in humans predominantly as a non-functional isoform (Kotnis et al., 2010). A single-nucleotide polymorphism leads to the canonical isoform which lacks 22 residues (328–349) encoded by exon 10, including the N-terminal (outer leaflet) portion of TM2 (Figure 2) (Le et al., 1997). Without a significant portion of TM2, this isoform is prone to subunit aggregation (Duckwitz et al., 2006). Restoration of the amino acids encoded by exon 10 is found to re-establish P2X_5_ function, producing strong currents in response to ATP (Bo et al., 2003). While, in humans, the allele encoding the non-functional protein is predominant, alleles encoding full-length, functional P2X_5_ have also been reported (Kotnis et al., 2010). Notably, the full-length, functional P2X_5_ isoform predominates in other species. Structures of the truncated and full-length isoforms might provide insight into the evolution of this P2XR subtype and the structural basis for the corresponding pharmacological effects.

While the sequence of hP2X_6_ appears to be quite similar to other P2XRs, this subtype lacks nine residues that compose the main portion of the left flipper, a key element in the activation of P2XRs (Figure 2) (Wang et al., 2017; Zhao et al., 2014; Li et al., 2010; Kawate, 2017; Jiang et al., 2012). These missing residues might explain why P2X_6_ homotrimers do not produce currents in response to ATP (Soto et al., 1996; Le et al., 1998; King et al., 2000). However, all residues that are known to coordinate ATP in other P2X subtypes are conserved within hP2X_6_ (Figure 2). This suggests that while ATP may be able to bind in the orthosteric binding site of hP2X_6_, the lack of a functional left flipper may be the cause of this subtype’s inability to transition to an ATP-bound open state. The functional significance of hP2X_6_ may be found in its ability to form heterotrimers, thereby expanding the pharmacological complexity of purinergic signaling (Le et al., 1998; King et al., 2000; Antonio et al., 2014).

Easily seen in the sequence alignment, P2X_7_ has several features that are specific to this subtype, including the C-cys anchor (residues 360–377) and its uniquely large cytoplasmic domain, referred to as the cytoplasmic ballast (Figure 2) (McCarthy et al., 2019). Post-translational palmitoylation of residues on the C-cys anchor explains the distinct ability of P2X_7_ to remain open without undergoing desensitization (McCarthy et al., 2019). Mapping the unique features of P2X_7_ from sequence to structure demonstrates the importance of resolving the cytoplasmic domains for each of the other P2XRs.

## Orthosteric Ligand Binding

**FIGURE 3:**
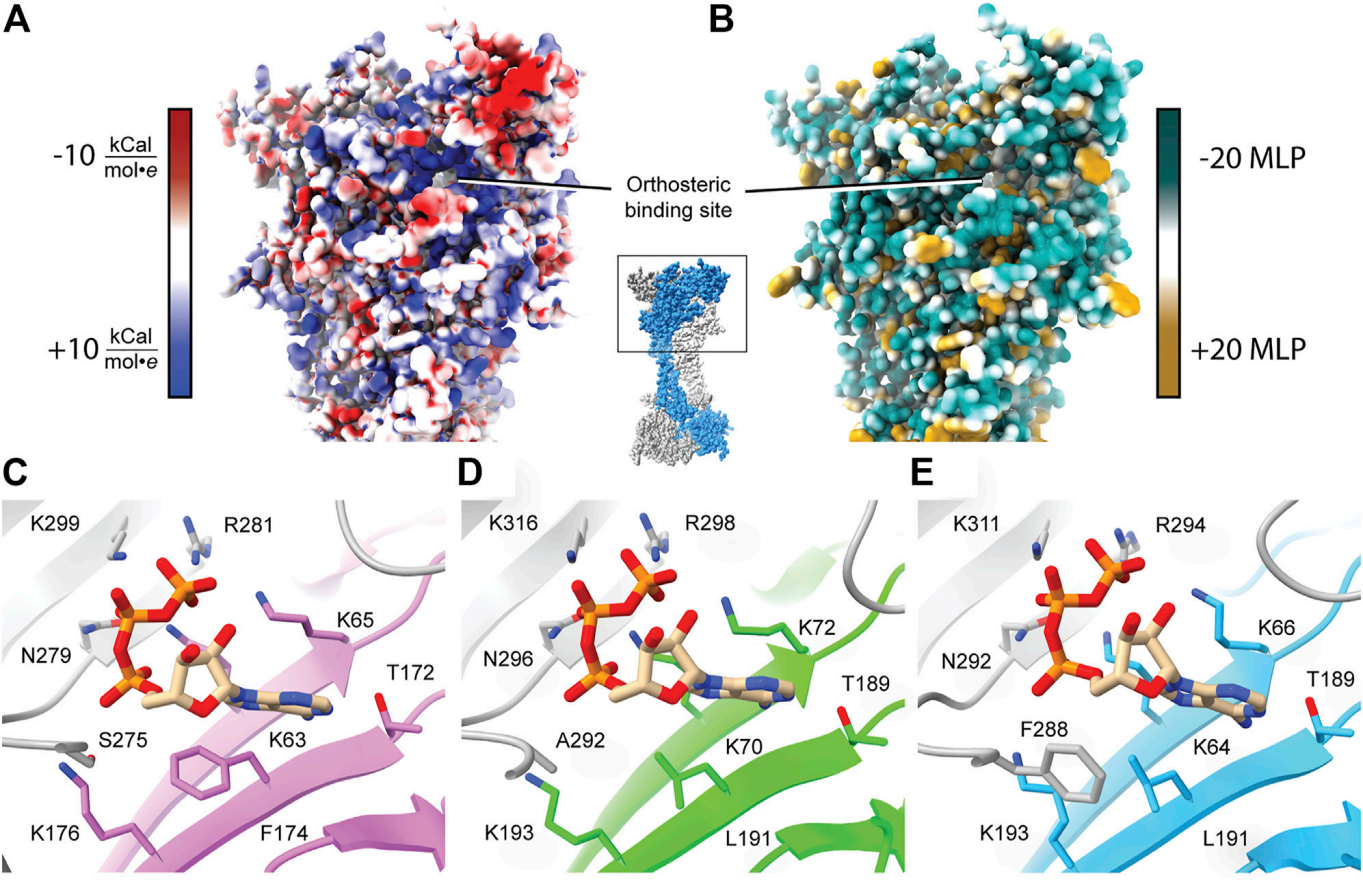
Characteristics of the orthosteric binding site of hP2X_3_, zfP2X_4_, and rP2X_7_. **(A)** Electrostatic surface rendering of rP2X_7_ (PDB: 6U9V) with blue and red coloring representing positive and negative electrostatic potential, respectively, highlighting the electropositive nature of the P2XR orthosteric ATP-binding site. **(B)** Hydrophobic surface rendering of rP2X_7_ (PDB: 6U9V) with hydrophobic regions colored brown and hydrophilic regions colored turquoise, each defined by positive and negative molecular lipophilicity potential (MLP), respectively (Laguerre et al., 1997). This rendering highlights the hydrophilic nature of the orthosteric ATP-binding pocket. **(C–E)** Conserved residues involved in the coordination of ATP in the orthosteric binding pocket across structurally characterized P2XRs. Panels are annotated with ortholog-specific residue numbering. Orthosteric site of **(C)** the ATP-bound open state hP2X_3_ structure (light purple, PDB: 5SVK), **(D)** the ATP-bound open state zfP2X_4_ structure (green, PDB: 4DW1), and **(E)** the ATP-bound open state rP2X_7_ structure (blue, PDB: 6U9W). All bound ATP molecules occupy a nearly identical U-shaped pose. The MLP and electrostatic surface potential were calculated in ChimeraX (Pettersen et al., 2021).

It is crucial to elucidate how ATP interacts with the orthosteric binding site in order to understand the mechanism and modulation of P2XR activation. One approach to inhibit P2XR function is to develop competitive antagonists that target the orthosteric ATP-binding site (Figure 1B). The residues that compose the orthosteric pocket are positively charged, hydrophilic, and highly conserved across all receptor subtypes (Figures 2, 3A,B) (Chataigneau et al., 2013). Similarity of the orthosteric site across P2XRs might impede the development of subtype-selective competitive antagonists. However, structural studies can provide crucial insights into the subtle differences in molecular pharmacology between P2XR subtypes at the orthosteric site.

The published structures of hP2X_3_, zfP2X_4_, and rP2X_7_ receptors in an ATP-bound open state define the orthosteric binding site (Figure 3). A close look at these structures reveals that ATP occupies a virtually identical U-shaped pose and the principal residues responsible for ATP coordination are highly conserved: four lysines, a threonine, an asparagine, and an arginine (Figures 2, 3C–E) (Hattori and Gouaux, 2012; Mansoor et al., 2016; McCarthy et al., 2019). Despite this conservation, the molecular pharmacology of P2XR activation by ATP is remarkably variable across subtypes. For example, P2X_7_ requires ∼200-fold higher concentration of eATP than P2X_3_ for activation (Jarvis and Khakh, 2009; Illes et al., 2021). A comparison of the ATP-bound open state structures of hP2X_3_, zfP2X_4_, and rP2X_7_ reveals there are differences in only two of the residues that directly coordinate ATP. The first difference is a hydrophobic residue that interacts with the adenosine base (F174, L191, and L191 in hP2X_3_, zfP2X_4_, and rP2X_7_, respectively) (Figures 2, 3). The second variable residue is on a loop within the left flipper which differs between a serine (S275) in hP2X_3_ that interacts with the α-phosphate of ATP, an alanine (A292) in zfP2X_4_ that makes no contacts to ATP, or a phenylalanine (F288) in rP2X_7_ that interacts with C5 on the ribose of ATP. These minor variations in the principal residues of the orthosteric binding site between subtypes seem unlikely to fully explain the dramatic differences in ATP sensitivity, suggesting there are other factors at play.

**FIGURE 4:**
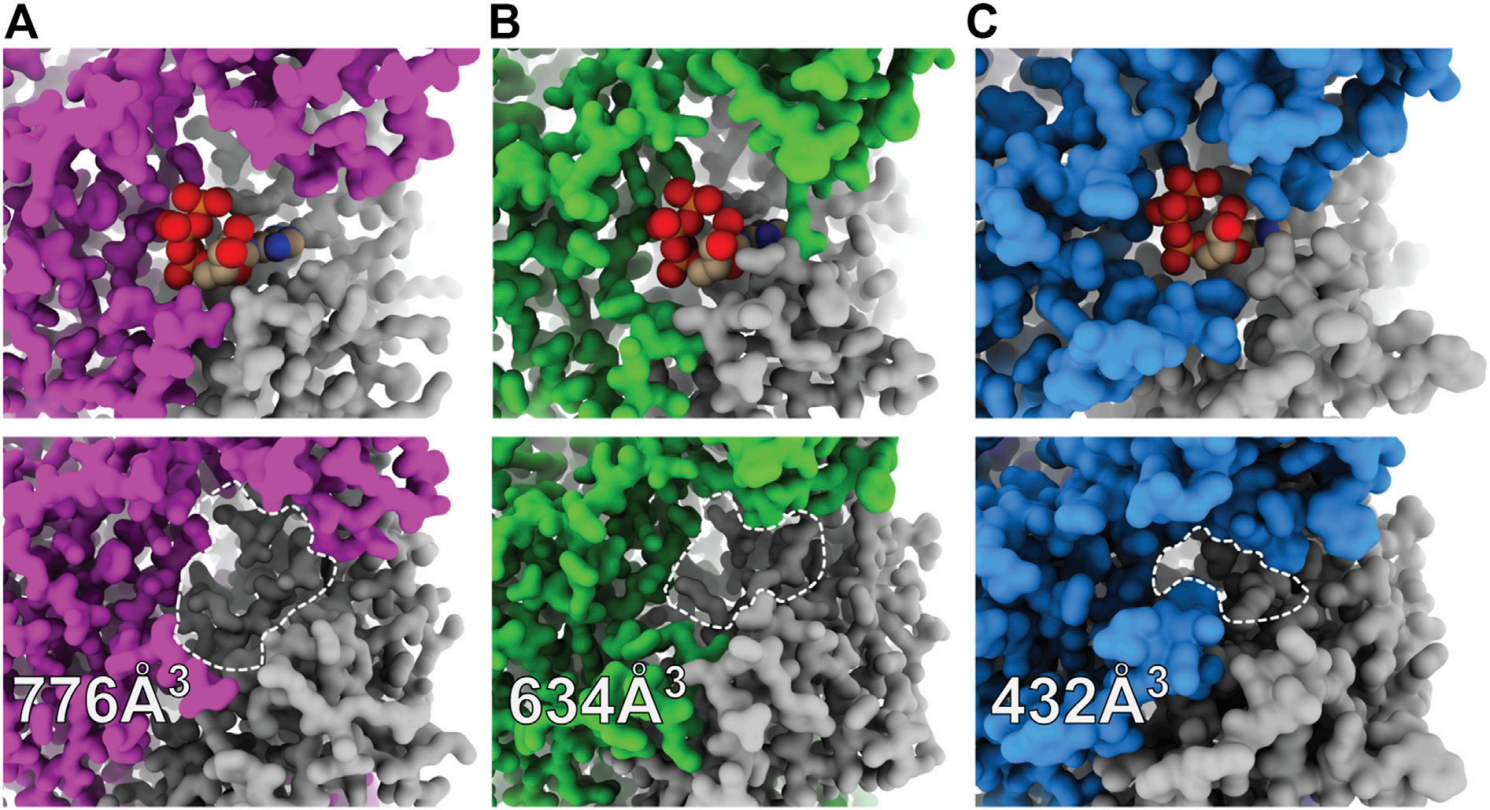
Surface representations of orthosteric ATP-binding pockets highlight differential ligand accessibility between P2XR subtypes. **(A)** ATP-bound open (top) and apo closed (bottom) states of hP2X_3_ (light purple, PDBs: 5SVK and 5SVJ) reveal the highly accessible pocket. **(B)** ATP-bound open (top) and apo closed (bottom) states of zfP2X_4_ (green, PDBs: 4DW1 and 4DW0) reveal a slightly less accessible pocket. **(C)** ATP-bound open (top) and apo closed (bottom) states of rP2X_7_ (blue, PDBs: 6U9W and 6U9V) reveal the least accessible pocket. The approximate binding pockets are outlined with dashed lines for visual clarity. Reported solvent accessible pocket volumes of the orthosteric ATP-binding site in the apo states of hP2X_3_, zfP2X_4_, and rP2X_7_ were calculated with CASTp (using a 2.0 Å probe radius) to be 776, 634, and 432 Å^3^, respectively (Tian et al., 2018).

A kinetic limitation to ATP-binding may be one such factor contributing to differential ATP sensitivities between P2XR subtypes. Solvent accessibility to the orthosteric binding site in the apo closed state structures of hP2X_3_, zfP2X_4_, and rP2X_7_ are strikingly different (Figure 4) (Stank et al., 2016; McCarthy et al., 2019). The surface-accessible volume of the orthosteric binding site in rP2X_7_ is approximately 44% smaller than in hP2X_3_ and 32% smaller than in zfP2X_4_ (Figure 4) (Tian et al., 2018). The effects of variable accessibility can perhaps be explained by small side-chain fluctuations and backbone or interdomain vibrational movements, referred to as “pocket breathing” (Ferrari et al., 2003; Stank et al., 2016). Local protein dynamics and flexibility would affect the kinetics of ligand binding by restricting pocket accessibility, thereby requiring higher concentrations of ATP to activate P2X_7_. The identity of the coordinating residues and the accessibility of the orthosteric site are valuable insights gained from structural analysis, both of which are necessary considerations during the design of subtype-selective antagonists.

The effect that divalent cations have on the activity of select P2XRs is another consideration for the difference in ATP sensitivities between subtypes. The presence of divalent cations in the extracellular environment shifts the activation requirements for P2X_2_, P2X_4_, and P2X_7_ to higher concentrations of ATP (Garcia-Guzman et al., 1997; Virginio et al., 1997; Li et al., 2013). In P2X_2_, the affinity and efficacy of MgATP^2-^ is lower than that of free ATP, suggesting Mg^2+^ acts as an inhibitor and constrains receptor function. In contrast, MgATP^2-^ effectively agonizes P2X_1_ and P2X_3_ at similar concentrations to free ATP (Li et al., 2013). The crystal structures of the apo closed state and ATP-bound open state of hP2X_3_ confirm that Mg^2+^ binds in two distinct conformations at an acidic chamber near the orthosteric binding site (Mansoor et al., 2016; Li et al., 2019). Functional data for hP2X_3_ suggest that Mg^2+^ slows ATP release and receptor recovery from desensitization (Li et al., 2019). However, direct equilibrium binding data demonstrate the presence of Mg^2+^ does not influence the affinity of hP2X_3_ for ATP (Mansoor et al., 2016). The subtype-specific effects of divalent cations on P2XR activation and gating need to be investigated further.

**FIGURE 5:**
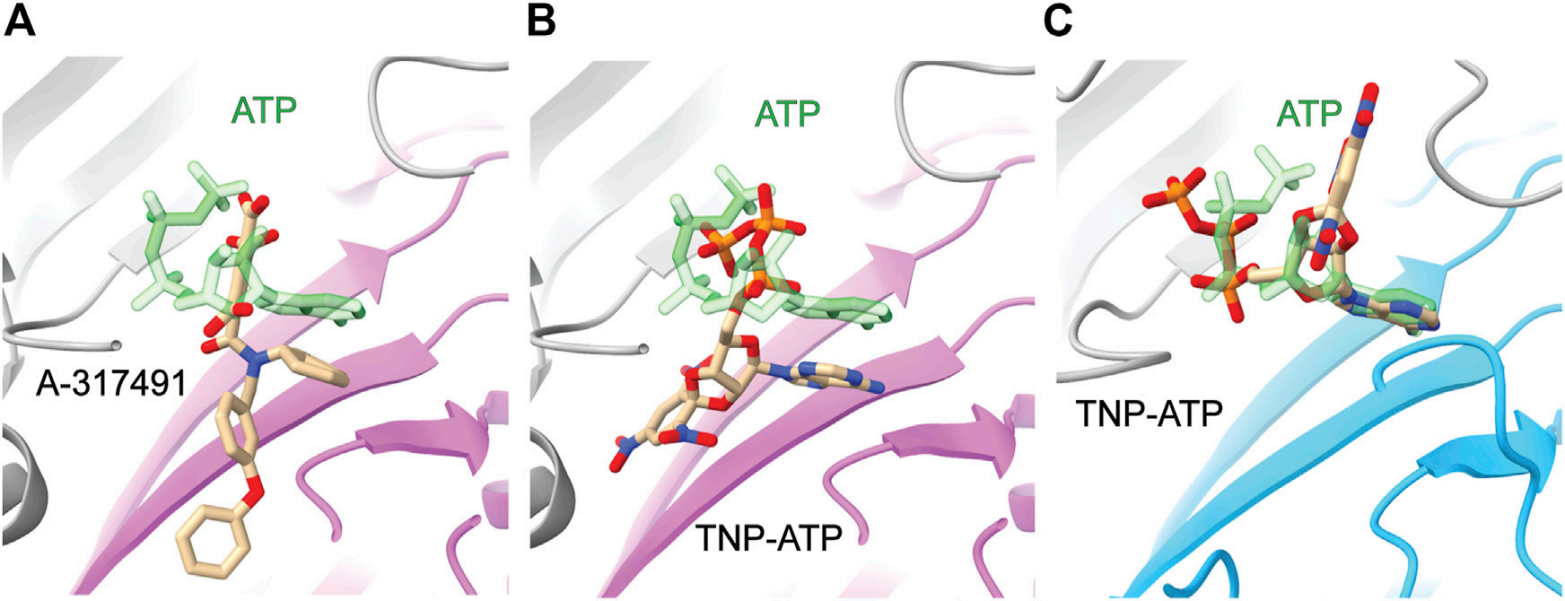
Comparative ligand poses of competitive antagonist-bound closed state and ATP-bound open state P2XR structures. **(A)** The Y-shaped pose of competitive antagonist A-317491 (tan) bound to hP2X_3_ contrasts with the U-shaped pose of ATP (green) bound to hP2X_3_ (purple). Superimposed PDBs: 5SVR and 5SVK. **(B)** The Y-shaped pose of competitive antagonist TNP-ATP (tan) bound to hP2X_3_ contrasts with the U-shaped pose of ATP (green) bound to hP2X_3_ (purple). Superimposed PDBs: 5SVQ and 5SVK. **(C)** The extended pose of competitive antagonist TNP-ATP (tan) bound to ckP2X_7_ contrasts with the U-shaped pose of ATP (green) bound to rP2X_7_ (blue). Superimposed PDBs: 5XW6 and 6U9W.

The information gained from the few known competitive antagonists that target P2XRs provides crucial insight into receptor modulation (Wolf et al., 2011; North and Jarvis, 2013; Muller, 2015; Muller and Namasivayam, 2021; Illes et al., 2021). The two structures of competitive antagonists bound to hP2X_3_ (TNP-ATP and A-317491) reveal these high-affinity antagonists bind at the orthosteric binding site in a Y-shaped pose, distinct from the U-shaped pose of ATP (Figures 5A,B) (Mansoor et al., 2016). This shift in ligand pose allows for deeper penetration into the binding pocket and is postulated to prevent the conformational changes necessary for channel opening (Mansoor et al., 2016). Residues D158 and F174 (hP2X_3_) are theorized to confer high specificity of TNP-ATP for hP2X_3_ and hP2X_1_, as this pair of key residues is only conserved in these two P2XR subtypes (Figure 2) (Mansoor et al., 2016). TNP-ATP occupies a different, extended conformational pose in the orthosteric binding site of ckP2X_7_, distinct from both the U-shaped pose of ATP in rP2X_7_ and Y-shaped pose of TNP-ATP in hP2X_3_ (Figures 5B,C) (Kasuya et al., 2017). The phosphate groups of TNP-ATP in ckP2X_7_ are stabilized in this extended conformation by hydrogen bond interactions from residues K236 and K298. Notably, while TNP-ATP inhibits P2X_1_ and P2X_3_ with low nanomolar affinity, hundreds of micromolar of TNP-ATP are required to antagonize P2X_7_ (Virginio et al., 1998). This drastic difference in apparent affinity and the distinct binding poses of TNP-ATP between hP2X_3_ and ckP2X_7_ may be correlated. The multitude of poses occupied by ATP, TNP-ATP, and A-317491 reveal the targetable space within the orthosteric pocket, providing knowledge that should be used in development of general and subtype-specific competitive antagonists. These structures reveal the importance of subtype-specific residues and pocket accessibility as critical factors for consideration in SBDD.

## Allosteric Ligand Binding

Identification of allosteric binding sites in P2XRs is crucial for the development of novel therapeutic ligands (Muller, 2015; Muller and Namasivayam, 2021). These sites bind secondary ligands non-competitively, promoting structural conformations that enhance or suppress orthosteric ligand binding. From a therapeutic perspective, allosteric modulators are advantageous as they often have fewer side effects compared to orthosteric ligands due to higher receptor specificity, resulting in less disruption of other functional pathways (Coddou et al., 2011; Nussinov and Tsai, 2012; Wenthur et al., 2014; Muller, 2015; Changeux and Christopoulos, 2016; Wang et al., 2018). This is particularly important when targeting a protein whose orthosteric ligand is ATP, a common substrate for proteins involved in metabolism and neurotransmission. Until recently, all known allosteric modulators against P2XRs were non-natural synthetic ligands. Current data now indicate bilirubin is an endogenous ligand that acts as a negative allosteric modulator of P2X_7_, suggesting the possibility for native cellular mechanisms of P2XR allosteric regulation (Zhao et al., 2021). It would be intriguing to discover other endogenous ligands that modulate P2XRs, determine their role in physiological or pathophysiological states, and adapt their chemical scaffold for the development of novel ligands. In support of the proposed therapeutic value of the P2XR allosteric sites, there are numerous small-molecule antagonists of P2XRs in various phases of clinical trials (Kamei et al., 2005; Pfizer, 2008; Keystone et al., 2012; Abdulqawi et al., 2015; Afferent Pharmaceuticals, 2015; CCTU-Core et al., 2019; Richards et al., 2019).

There are currently two distinct allosteric binding sites within P2XRs that have been confirmed with high-resolution structures, one near the orthosteric binding site and another on the top of the extracellular domain, visualized in hP2X_3_ and pdP2X_7_, respectively (Figures 1B,C) (Karasawa and Kawate, 2016; Wang et al., 2018). Electrophysiological and biochemical studies additionally show allosteric sites exist in P2X_1_ and P2X_4_ (Muller, 2015; Ase et al., 2019; Obrecht et al., 2019; Illes et al., 2021; Muller and Namasivayam, 2021; Bidula et al., 2022). Sequence analysis further suggests the remaining P2XRs also contain allosteric sites (Figure 2). Since each distinct allosteric site exists at the interface of two protomers, it is unclear to what extent similar allosteric sites exist within heterotrimeric receptors where the interface between subtypes might be markedly different. Examination of the available P2XR structures in complex with allosteric modulators provides pivotal insights into the regulation of P2XRs.

**FIGURE 6:**
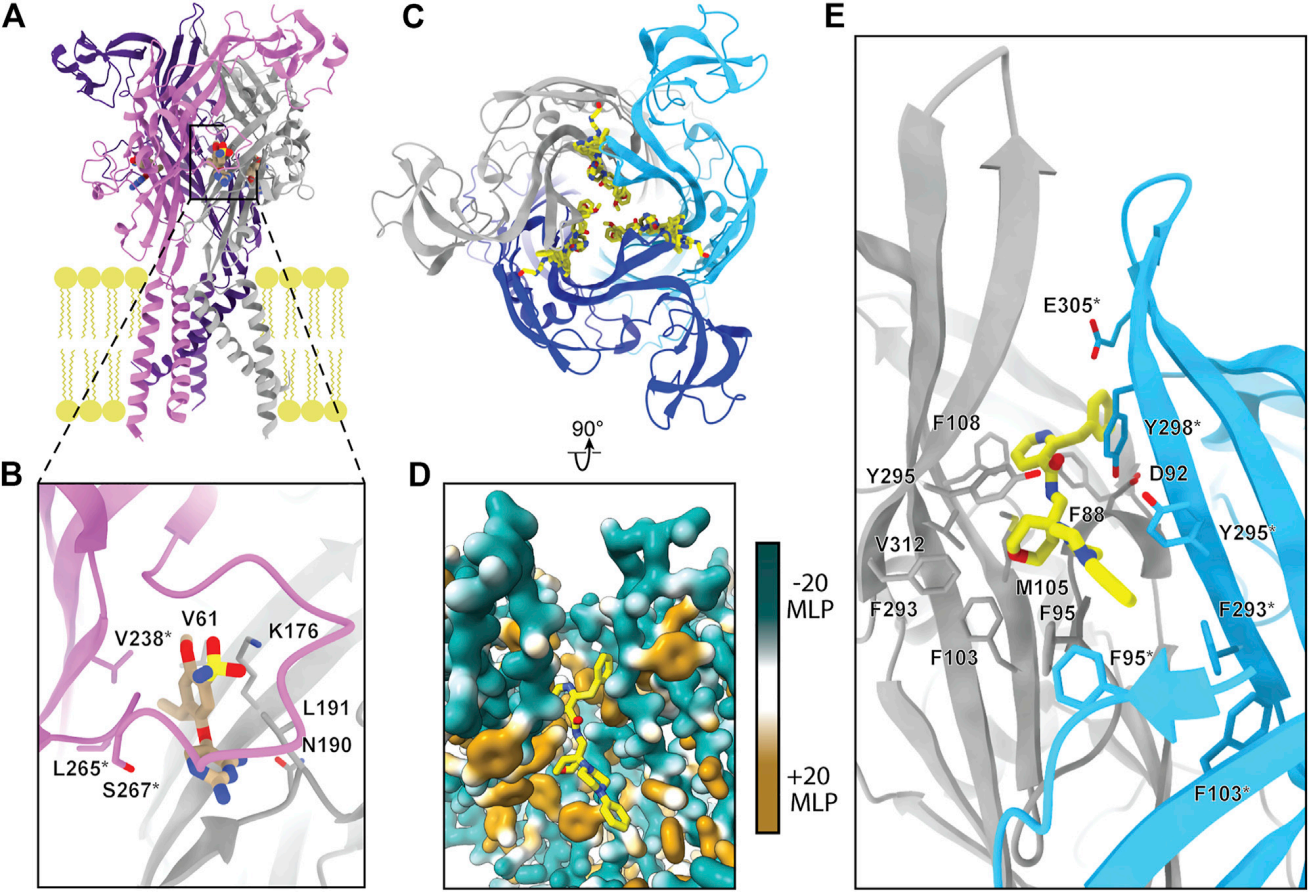
The two known allosteric sites of P2XRs. **(A)** Ribbon representation of the negative allosteric modulator gefapixant (tan) bound to hP2X_3_ (PDB: 5YVE). **(B)** Magnified view of panel A showing gefapixant interacting with residues at the interface of the left flipper of one protomer (purple) and lower body of another (grey) in hP2X_3_. **(C)** Top-down view of five superimposed negative allosteric modulators (yellow) bound to a ribbon representation of pdP2X_7_, highlighting the nearly identical binding mode of the chemically distinct ligands (PDBs: 5U1W, 5U1X, 5U1Y, 5U1V, and 5U1U). **(D)** Magnified and rotated view of panel C showing one of the five negative allosteric modulators (JNJ-47965567) bound to the hydrophobic pocket of pdP2X_7_ (PDB: 5U1X). Hydrophobic surface rendering of pdP2X_7_ with hydrophobic regions colored brown and hydrophilic regions colored turquoise, each defined by positive and negative molecular lipophilicity potential (MLP), respectively (Laguerre et al., 1997). **(E)** Ribbon representation of panel D with negative allosteric modulator JNJ-47965567 bound to pdP2X_7_ highlighting interacting residues (PDB: 5U1X). *Demarcating residues in the purple and blue protomers in panel B and in panel E, respectively. The MLP was calculated in ChimeraX (Pettersen et al., 2021).

One of the visualized allosteric sites in P2XRs was revealed in the structure of hP2X_3_ bound to the nanomolar-affinity negative allosteric modulator gefapixant (Wang et al., 2018). This site is composed of residues at a protomer interface formed by the left flipper of one subunit and the lower body and dorsal fin of another (Figures 1A,B, 6A,B). This allosteric site is located on the opposite side of the left flipper as the orthosteric binding site. Movement of the left flipper is an essential step in the transition to an ATP-bound open state (Wang et al., 2017; Zhao et al., 2014; Li et al., 2010; Kawate, 2017; Jiang et al., 2012). Negative allosteric modulators that bind this site, including gefapixant, restrict left flipper mobility and thereby inhibit channel opening. Analysis of the gefapixant-bound hP2X_3_ structure indicates that residue K176, which is fully conserved across all human P2XRs, is of particular importance (Figure 2). It is the only residue in common between this allosteric site and the orthosteric ATP-binding site, interacting with the two oxygens of the sulfonyl nitrene moiety of gefapixant or with an oxygen on the α-phosphate of ATP (Figures 3C, 6B). More biochemical experimentation is required to determine if both ligands can occupy their respective binding sites simultaneously. Several other residues interacting with gefapixant in hP2X_3_ are of note; N190 is fully conserved and L191, V238, and L265 display hydrophobicity across all P2XR subtypes (Figures 2, 6B). Interestingly, all residues in this allosteric site (except V61 and S267) are conserved between hP2X_3_ and hP2X_1_, suggesting this allosteric site is present and quite similar in both subtypes. To support this idea, the ligand aurintricarboxylic acid (ATA) is a high-affinity negative allosteric modulator of hP2X_1_ and hP2X_3_ (proposed to bind the same site as gefapixant), but does not effectively antagonize other subtypes (Obrecht et al., 2019). The characterization of this allosteric site within P2X_3_ (and potentially P2X_1_) provides a structural template for the development of P2XR subtype-specific antagonists to this novel site.

Crystallographic structures of five unique negative allosteric modulators bound to pdP2X_7_ provide a view of another allosteric site present in P2XRs (Karasawa and Kawate, 2016). Located on the top of the extracellular domain, ligands bound at this allosteric site prevent a conformational rearrangement required for all P2XRs to undergo transition to an ATP-bound open state (Figures 1C, 6C) (Karasawa and Kawate, 2016; Kawate, 2017). This binding pocket is generally hydrophobic and the entrance is lined by charged residues (Figures 6D,E). The five negative allosteric modulators structurally characterized in complex with pdP2X_7_, despite varying in size, share a common scaffold that generally complements the properties of the pocket, with a mostly hydrophobic tail and a hydrophilic head connected through a narrow, nitrogenous linker (Figure 6D) (Karasawa and Kawate, 2016). The residues at this allosteric binding pocket of pdP2X_7_ are similar or conserved in hP2X_7_ and across other human P2XRs, suggesting this site may be present in other subtypes. In support of this, the proposed negative allosteric modulator 5-(3-Bromophenyl)-1,3-dihydro-2*H*-benzofuro [3,2-*e*]-1,4-diazepin-2-one (5-BDBD) is reported to bind to P2X_4_ at the equivalent allosteric site (Bidula et al., 2022). Altogether, the structures of allosteric modulators bound to pdP2X_7_ and the biochemical experiments on P2X_4_ suggest this site is a promising target for the development of novel high-affinity and subtype-specific P2XR antagonists by SBDD.

## P2XR Pore Architecture

**FIGURE 7:**
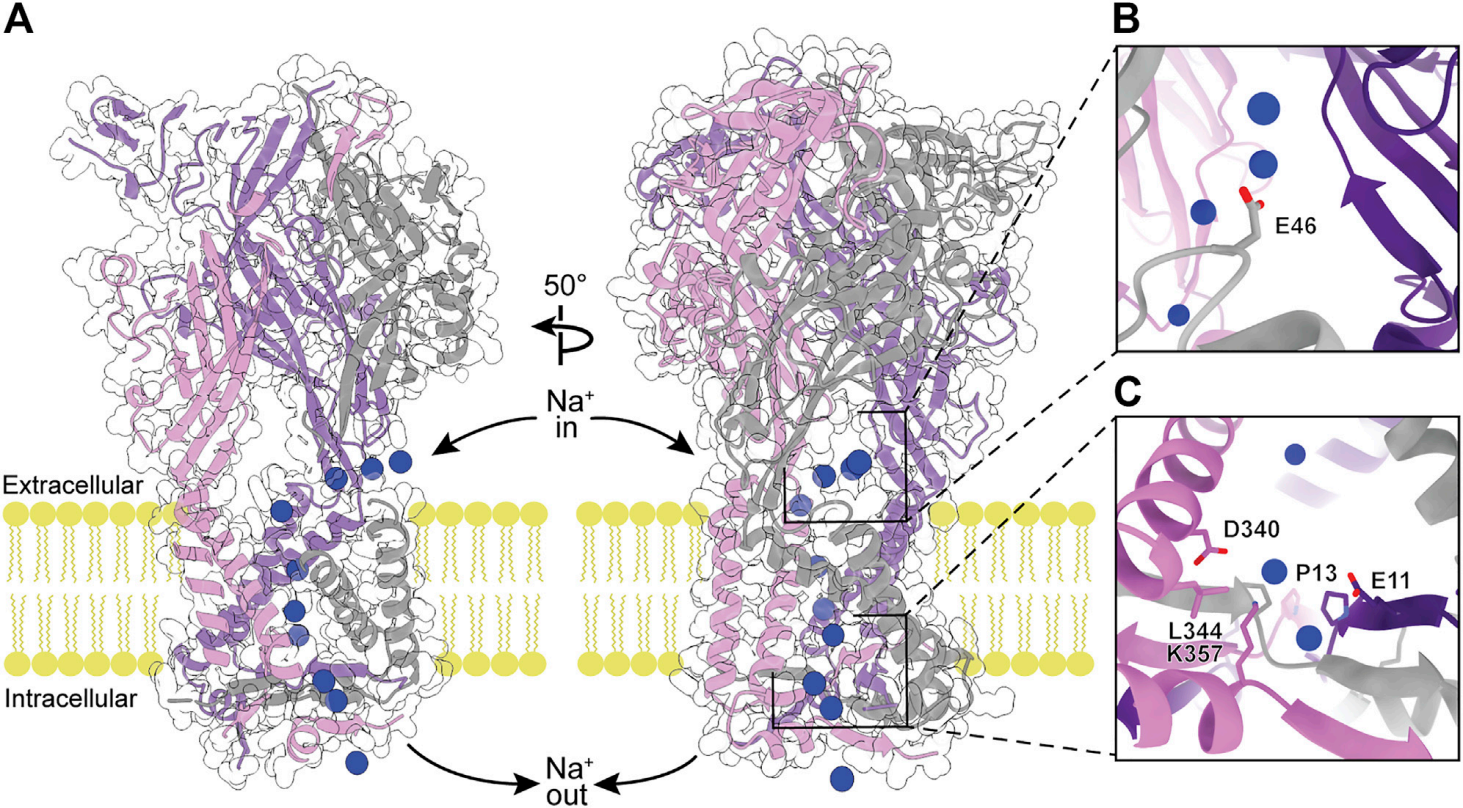
The modeled flow of Na^+^ ions through the ATP-bound open state hP2X_3_ structure (PDB: 5SVK). **(A)** Molecular dynamic simulations show Na^+^ ions (blue spheres) enter the receptor through the extracellular fenestrations, pass through the extracellular vestibule and the pore, and enter the cell via the cytoplasmic fenestrations (Mansoor et al., 2016). *Left:* View highlighting the flow of Na^+^ ions through the pore. *Right:* 50^o^ rotated view highlighting the flow of Na^+^ ions through extracellular and cytoplasmic fenestrations. **(B)** Magnified view of Na^+^-coordinating residue (E46) within the extracellular fenestration. **(C)** Magnified view of key residues in the cytoplasmic fenestration. Mutations T13P/S15V/V16I were needed to slow the rate of desensitization and capture the ATP-bound open state hP2X_3_ crystal structure (Hausmann et al., 2014).

**FIGURE 8:**
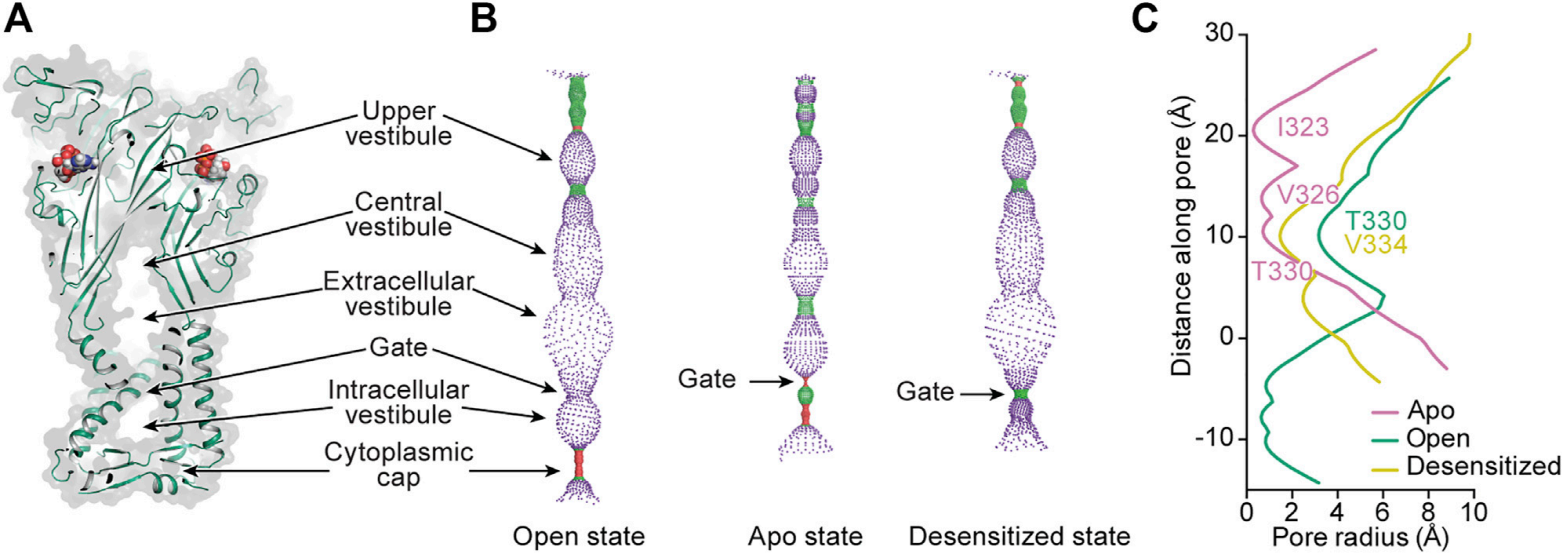
Mapping the interior space of the hP2X_3_ pore in different conformational states. **(A)** A coronal section of a surface representation of the ATP-bound open state of hP2X_3_ (PDB: 5SVK) reveals the upper, central, extracellular, and intracellular vestibules.**(B)** Pore-lining surface along the axis of hP2X_3_ for the ATP-bound open, apo closed, and ATP-bound desensitized states (PDBs: 5SVK, 5SVJ, and 5SVL, respectively), calculated by the program HOLE: red <1.15 Å radius, green between 1.15–2.30 Å radius, and purple >2.30 Å radius (Smart et al., 1996). **(C)** Plot of pore radius as a function of distance along the pore axis for the ATP-bound open, apo closed, and ATP-bound desensitized states of hP2X_3_. The positions of the residues making up the narrowest radius in each conformational state are labeled. The Cα position of I341 in hP2X_3_ is set as zero. This figure was from adapted from Mansoor et al. (2016).

As the primary function of P2XRs is to serve as non-selective cation channels in response to activation by eATP, another approach to inhibit ion channel function is by obstructing the ion path through the protein. The pore through the transmembrane region of P2XRs is formed by a bundle of TM helices in a structure similar to those found in acid-sensing ion channels (ASICs) and epithelial Na^+^ channels (ENaCs) (Noreng et al., 2018; Jasti et al., 2007; Baconguis et al., 2014). Ions flow through lateral, phospholipid-lined fenestrations into vestibules on both sides of the TM helices (Figures 1, 7, 8) (Samways et al., 2011; Samways et al., 2014). To validate the ion flow path, hP2X_3_ was crystallized in the presence of CsCl and probed for the location of Cs^+^ ions by anomalous signal (Mansoor et al., 2016). Glutamate E46 was found to coordinate a Cs^+^ ion at the entrance to each of the three extracellular fenestrations, indicating this residue similarly coordinates Na^+^ ions under physiological conditions (Figure 7B). The residue at this position is a glutamate or aspartate in four of the human subtypes (P2X_1_, P2X_3_, P2X_4_, and P2X_7_), but a glutamine in hP2X_2_ and a lysine in hP2X_5_ and hP2X_6_ (Figure 2). In hP2X_2_, hP2X_5_, and hP2X_6_, this ion-coordinating role in the fenestrations is likely fulfilled by a nearby acidic residue on an alternate protomer (E69, D57, or E66, respectively). This conserved charge at the lateral extracellular fenestrations could be targeted by designing ligands that prevent ion entry, regardless of whether the pore is open or closed. Such a ligand would need to selectively bind and block each extracellular fenestration, which are large surface domains with substantial void space in the ATP-bound open state (Figures 1B, 7A). This may be one instance where P2XR modulation is best achieved by a larger biologic therapeutic, such as an antibody or an aptamer (Nelson et al., 2010; Zhou and Rossi, 2017). These structured protein and nucleic acid affinity reagents are ideally suited for targeting large extracellular epitopes, where accessing small binding pockets is not necessary and diffusion across the cell membrane is not desired.

Ions travel from one of the extracellular fenestrations to the extracellular/central vestibule lined by the first β-strand after TM1 and the last β-strand before TM2 (Figures 1A, 8A,B). Through molecular modeling and mutagenesis studies, this large vestibule has been found to be a binding site for ginsenoside (“steroid-like” dammarane triterpenoid glycosides) allosteric modulators of hP2X_7_ (Bidula et al., 2019). Simulations suggest these molecules bind in the upper part of this vestibule (central vestibule), making key interactions to the β-strand immediately preceding TM2. While no structures of ginsenoside molecules bound to P2XRs exist at this time, this site may prove to be an attractive target for new allosteric modulators due to its location near the transmembrane pore. The upper vestibule is also the location of a Zn^2+^-binding site seen in the tick P2X structure (AmP2X) (Kasuya et al., 2016). Of the human subtypes, only hP2X_4_ has residues (Q94, E95) similar to the Zn^2+^-coordinating residues (E105, E106) of AmP2X (Figure 2). The activity of hP2X_4_ has been shown to be modulated by Zn^2+^, an interesting consideration for development of P2X_4_-specific ligands (Garcia-Guzman et al., 1997).

From the extracellular/central vestibule, ions cross the membrane through a channel (the pore) at the symmetry axis formed by a set of six TM helices consisting of two antiparallel helices from each protomer (Figures 1, 8A). In each protomer, the N-terminal α-helix (TM1) is oriented from cytosolic to extracellular and TM2 is oriented oppositely, forming a short section of antiparallel coiled-coil. The interior of the bundle is formed by TM2 from each protomer. The pore of the apo closed state is gated towards the extracellular side of the transmembrane region, with minimum radii of 0.3 Å at symmetry-related I323 in hP2X_3_ and 0.1 Å at S339 in rP2X_7_ (Figures 8B,C). Interestingly, these residues are on different turns of TM2 in the superimposed structures, showing there is some variability in the placement of the extracellular gate between subtypes. Upon the binding of ATP, movement of the three TM2 helices opens the channel to a pore radius large enough for passage of partially hydrated sodium ions (minimum radii of 3.2 Å in hP2X_3_ and 2.5 Å in rP2X_7_) (Degrève et al., 1996). Desensitization of P2X_3_, and presumably other P2XRs (except P2X_7_ which does not desensitize), occurs through another movement of the TM2 helices, whereby disassembly of the cytoplasmic cap leads to a new gate near the center of the transmembrane region at V334 in hP2X_3_ (radius of 1.5 Å). This new gate is too narrow to allow passage of hydrated sodium ions (Figures 8B,C) (Mansoor et al., 2016).

ASICs and ENaCs have known pore-blocking molecules such as amiloride, which binds near the extracellular end of the TM2 helices (PDB: 4NTX) (Kleyman and Cragoe, 1988; Baconguis et al., 2014). Considering the structural similarities between ASICs, ENaCs, and P2XRs, it is conceivable that amiloride derivatives or other molecules might also be found to target this region in P2XRs. Deeper areas of the pore, elucidated by cryo-EM, could also prove to be attractive drug targets despite being less accessible to extracellular molecules.

At the cytosolic end of the P2XR pore, the cytoplasmic cap forces the ions to egress laterally through the three cytoplasmic fenestrations (Figures 7A,C) (Mansoor et al., 2016). These fenestrations are roughly triangular in shape, with two sides formed by the ends of TM1 and TM2 from adjacent protomers and the third (distal) side formed by part of the cytoplasmic cap. The distal sides of the fenestrations are lined with charged side chains from each of the protomers, providing a favorable path for ions to exit the pore and enter the cell. The residues D340 and K357 form a salt bridge in the cytoplasmic fenestration of hP2X_3_ (Mansoor et al., 2016). Of note, these two residues are absolutely conserved across human subtypes while there is diversity in the other residues of the fenestrations (Figures 2, 7C). As in the extracellular fenestrations, these conserved charges may be useful in designing drugs to target the cytosolic fenestrations.

The P2XR ion path contains several locations that may prove to be attractive drug targets, including the extracellular and cytoplasmic fenestrations, the extracellular vestibule, and the transmembrane portion of the pore. The existing P2XR structures provide starting points for SBDD. Structures of the other human P2XRs would prove extremely useful in designing ligands that effectively and specifically target the pore.

## P2XR Cytoplasmic C-Terminal Domains

**FIGURE 9:**
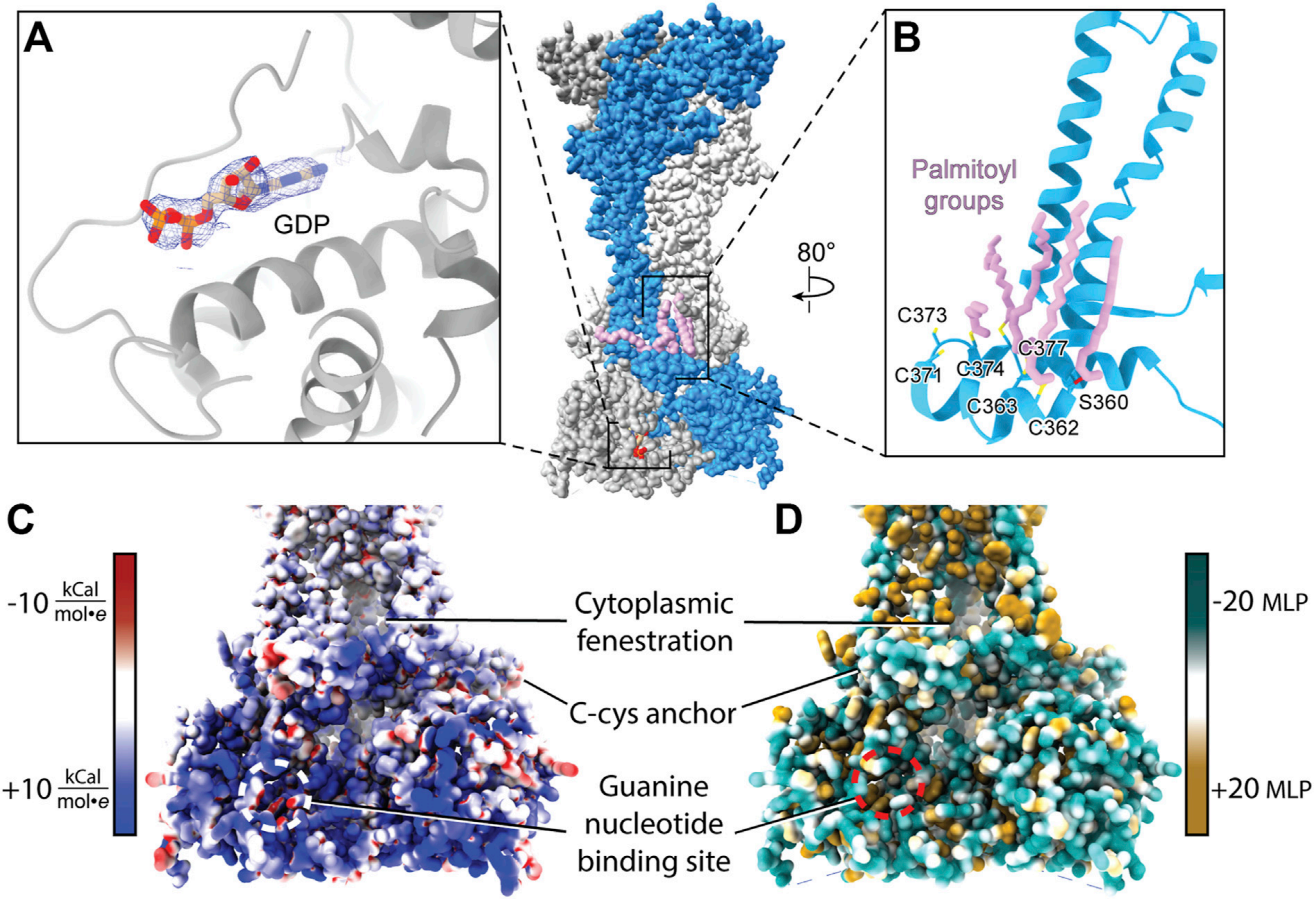
Attributes of the cytoplasmic domain of rP2X_7_ (PDB: 6U9V). **(A)** GDP bound to the cytoplasmic ballast of rP2X_7_ with its cryo-EM density shown in blue mesh (EMDB: 20702). **(B)** The C-cys anchor of rP2X_7_ contains palmitoyl groups (light purple) that extend into the membrane and prevent desensitization. **(C)** Electrostatic surface rendering of the rP2X_7_ cytoplasmic domain (PDB: 6U9V) with blue and red coloring representing positive and negative electrostatic potential, respectively, highlighting the electropositive nature of the cytoplasmic ballast. **(D)** Hydrophobic surface rendering of the rP2X_7_ cytoplasmic domain with hydrophobic regions colored brown and hydrophilic regions colored turquoise, each defined by positive and negative molecular lipophilicity potential (MLP), respectively, highlighting the hydrophilic nature of the cytoplasmic ballast (Laguerre et al., 1997). The location of the guanine nucleotide-binding site is outlined in white and red in panel C and in panel D, respectively. The MLP and electrostatic surface potential were calculated in ChimeraX (Pettersen et al., 2021).

P2XR C-terminal domains are divergent in sequence and size and their impacts on receptor function remain poorly understood. These domains dramatically vary from approximately 3%–34% of total protomer length, corresponding to 12 residues per protomer for hP2X_4_ and 203 residues per protomer for hP2X_7_, respectively (Figure 2). Unfortunately, the full C-terminal domains were not present in any of the constructs used to obtain crystal structures (Mansoor et al., 2016; Hattori and Gouaux, 2012; Kawate et al., 2009; Karasawa and Kawate, 2016; Kasuya et al., 2017; Wang et al., 2018). The recent full-length cryo-EM structures of rP2X_7_ contain the only structurally characterized P2XR cytoplasmic domain beyond the cytoplasmic cap, including the subtype-unique C-cys anchor and cytoplasmic ballast (Figure 9) (McCarthy et al., 2019).

Within each protomer of P2X_7_ is a C-cys anchor which contains six cysteines and one serine that can be palmitoylated (Figure 9B). The palmitoyl moieties extend into the inner leaflet of the membrane, preventing the helical recoil movement of TM2 that would otherwise result in channel desensitization (McCarthy et al., 2019). When the C-cys anchor is removed or mutated to prevent palmitoylation, rP2X_7_ desensitizes similarly to each of the other P2XR subtypes (McCarthy et al., 2019). While the role of the C-cys anchor in the gating cycle has been characterized, details surrounding post-translational modifications to this element remain unclear. For example, it is currently unknown in what stage of folding or trafficking the palmitoyl groups are added, nor which specific palmitoyl acyl transferase (PAT) enzyme or adapter protein is responsible for this post-translational modification. PATs generally target specific substrates and are localized to the ER, the Golgi, the plasma membrane, and endosomes, providing precise spatial regulation to their activity (Charollais and Van Der Goot, 2009). Modifications to the palmitoylation of P2X_7_ have a dramatic effect on receptor gating and would presumably alter downstream signaling (Allsopp and Evans, 2015). As a result, modulating C-cys anchor palmitoylation poses as an interesting therapeutic avenue to pursue, despite the significant challenges. As with any drug that has an intracellular target, the membrane is a barrier to delivery (Aungst, 1993; Di and Kerns, 2003; Naylor et al., 2018). Given that many other proteins are also palmitoylated, selectively preventing P2X_7_ palmitoylation would require determining and specifically targeting the correct PAT(s) at the appropriate point in post-translational processing. A greater understanding of the basic biology of P2X_7_ maturation is necessary before such a pharmacological strategy can be attempted.

The discovery of a nanomolar-affinity guanine nucleotide-binding site in the cytoplasmic ballast of rP2X_7_, the residues of which are conserved in hP2X_7_, is quite intriguing (Figure 9A). While only GDP is visualized in the apo closed and ATP-bound open state rP2X_7_ structures following purification, both GTP and GDP were shown to bind with equally high affinity (McCarthy et al., 2019). The pose of GDP is such that the ribose and nucleobase are positioned internally and the phosphate tail is on the periphery of the cytoplasmic ballast and easily accessible to the intracellular environment and potential accessory proteins (Figure 9A). The guanine nucleotide-binding site is electropositive, hydrophilic, and exposed, making it an accessible docking site for protein-protein interactions (Figures 9C,D). It would be interesting if P2X_7_, like G-protein coupled receptors (GPCRs), has associated guanine nucleotide exchange factors (GEFs) that facilitate GDP release (Cherfils and Zeghouf, 2013). The presence of the cytoplasmic ballast is necessary for the metabotropic properties of P2X_7_, including the release of cytokines as well as activation of various lipases and kinases (El-Moatassim and Dubyak, 1992; Humphreys and Dubyak, 1996; Surprenant et al., 1996; Wilson et al., 2002; Cheewatrakoolpong et al., 2005; Costa-Junior et al., 2011; Kopp et al., 2019). While there is no direct data regarding a role of the guanine nucleotide-binding site in P2X_7_ signaling, it is intriguing to imagine the metabotropic signaling properties of the cytoplasmic ballast are dependent on guanine nucleotide binding. If, similar to GPCRs, guanine nucleotide binding proves to be critical for P2X_7_ signaling, then guanine nucleotide analog ligands (such as abacavir, acyclovir, and entecavir) may be applied to study and therapeutically target P2X_7_-mediated signaling (Seley-Radtke and Yates, 2018). As with targeting the orthosteric ATP-binding site, targeting intracellular guanine nucleotide-binding sites must be done carefully as GTP and GDP are crucial metabolic and signaling molecules for numerous biological processes. Altogether, cryo-EM facilitated the study of full-length rP2X_7_ and thus revealed the cytoplasmic domain in its entirety and the therapeutic potential it holds. With the unique cytoplasmic domains of the remaining six P2XRs yet to be structurally characterized, there are more avenues for novel subtype-specific therapeutic development to be discovered.

## P2XR Structural Elucidation by Cryo-EM

The recent structures of full-length rP2X_7_ facilitated key discoveries that were exclusively enabled by cryo-EM. The guanine nucleotide-binding site was identified only after the cryo-EM map revealed an unexplained non-protein density of sufficient quality to accurately predict ligand identity without *a priori* knowledge of its existence (Figure 9A) (McCarthy et al., 2019). The identity of the guanine nucleotide was subsequently validated to be GDP by mass spectrometry. The cryo-EM reconstructions similarly revealed the precise location of the palmitoylated residues in the C-cys anchor (including the unexpected palmitoylation of a serine residue), a task that proved to be challenging by other methods (Gonnord et al., 2009). Altogether, the discoveries empowered by cryo-EM have unveiled novel elements that will further our understanding of the biological functions of P2X_7_.

There remain four homotrimeric P2XR subtypes without published structures: P2X_1_, P2X_2_, P2X_5_, and P2X_6_. Given that these receptors are implicated in a wide range of physiological and pathophysiological states, it is crucial to understand the molecular pharmacology of each receptor subtype (Burnstock and Kennedy, 2011; North and Jarvis, 2013; Burnstock and Knight, 2018; Lara et al., 2020). Obtaining cryo-EM structures of these receptors will not only reveal their subtype-specific features but also facilitate SBDD to improve ligand selectivity. It is important to note that while homology modeling and machine-learning programs like AlphaFold are improving, there is no replacement for the certainty afforded by empirically determined ligand-bound or protein-protein complexed structures (Jumper et al., 2021). To this point, there are also many known P2XR ligands for which we do not know how (or even where) they bind receptors. These ligands dramatically vary in size, shape, and chemical identity—even between ligands known to be selective for a specific receptor subtype—suggesting there are additional uncharacterized allosteric sites present in P2XRs. Structures of each receptor subtype in complex with these different ligands will help define the molecular pharmacology of P2XRs and confirm the locations of novel allosteric sites.

To date, the published P2XR structures are predominantly of non-human orthologs. While these structures are helpful and enable more accurate homology models, it is known that P2XR orthologs have unique pharmacological features which can significantly impact drug development. For example, human, mouse, and rat P2X_7_ dramatically differ in response to known allosteric modulators. The negative allosteric modulator AZ11645373 is a high-affinity antagonist for hP2X_7_, yet is ∼100-fold less effective against mouse P2X_7_ (mP2X_7_) and >500-fold less effective against rP2X_7_ (Stokes et al., 2006; Michel et al., 2009). There are also negative allosteric modulators such as A-438079 and A-740003 that antagonize all three of these P2X_7_ orthologs with similar efficacy (Donnelly-Roberts et al., 2009). Therefore, using cryo-EM to understand the structural basis for the pharmacologic variability across P2XR orthologs is crucial for the development of novel therapeutics that effectively target human P2XRs.

**FIGURE 10:**
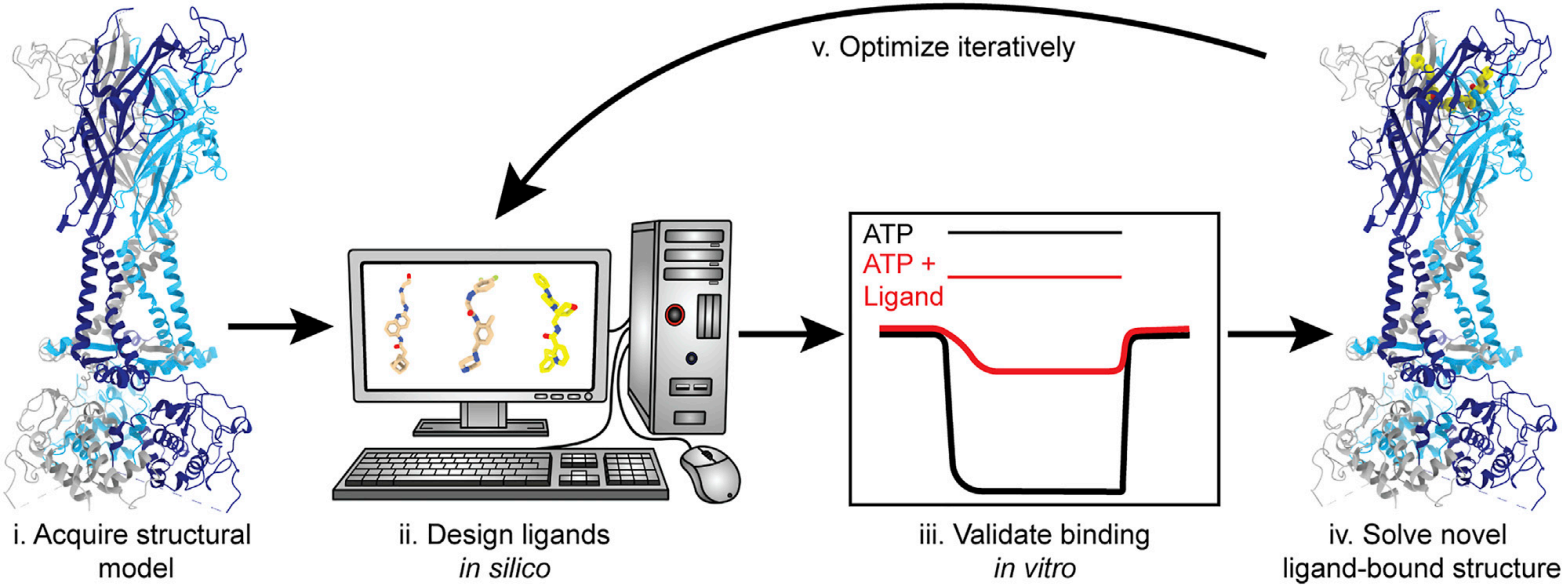
Structure-based drug design (SBDD) workflow using P2XRs as an example. SBDD includes five broad steps. (i) Solve receptor structure to high-resolution or use a reliable theoretical model to identify and define ligand-binding sites; (ii) Use molecular docking and modeling software (*in silico*) to identify and optimize small molecules capable of occupying a binding pocket; (iii) Chemically synthesize the most promising *in silico* “hits” and validate binding and selectivity by biochemical and electrophysiological methods; (iv) Solve the structure of the ligand-bound receptor complex empirically; and (v) Iterate steps (ii–iv) until ligand(s) with satisfactory properties are obtained and fully characterized. Representative structures generated from PDBs: 6U9V (for protein backbone) and 5U1X (for the bound ligand).

The feasibility of SBDD has dramatically increased with access to more advanced computational tools and the availability of structures for therapeutically relevant membrane proteins, largely due to the cryo-EM resolution revolution (Kuhlbrandt, 2014; Lionta et al., 2014; Lees et al., 2021). With the information gained from the currently published P2XR structures, SBDD can facilitate the discovery of ligands that target this receptor family (Figure 10). Molecular docking and *in silico* screening can probe any of the targetable sites within P2XRs for potential “hit” compounds (Ballante et al., 2021). Then, structure-based modeling can be used to optimize chemical groups, adding or substituting different moieties to improve affinity and specificity. Once computationally optimized, a ligand can be synthesized, evaluated *in vitro*, and (if validated) structurally solved in complex with its target receptor to high-resolution. This process will be repeated iteratively until a threshold of affinity and specificity is reached (Figure 10). Even though the use of cryo-EM has led to a dramatic number of new structures, the application of SBDD driven by this structural method is still an emerging area of research. Some recent successful cases that exemplify this approach include ligands that specifically target the TRPV5 channel, µ-opioid receptor, and the 80S ribosome (Wong et al., 2017; Hughes et al., 2019; Wang et al., 2022). Cryo-EM is currently the best method to solve ligand-bound P2XR structures and empower the use of SBDD.

## Concluding Remarks

The substantial body of work characterizing P2XR structures has provided invaluable information on the biology and molecular pharmacology for this receptor family, however there remain many unanswered questions. Little is known about the structure and function of P2XR cytoplasmic domains and even less about heterotrimeric P2XRs. While the structural determination of the P2X_7_ cytoplasmic domain provided valuable insights, unexpected findings raised even more questions about its biological function. Most importantly, we have yet to fully understand how to selectively modulate the seven homotrimeric P2XRs or how effective such modulators would be against heterotrimeric P2XRs, considering their pharmacological complexity. The recent advancements in cryo-EM make this technique ideally suited to study P2XRs, including novel receptor structures and ligand-bound receptor complexes. While P2XRs are implicated in a host of physiological and pathophysiological conditions found within the cardiovascular, central nervous, and immune systems, there are no FDA-approved drugs targeting this receptor family. The success of antiplatelet drugs which antagonize the related P2Y GPCR family exemplifies the therapeutic value of targeting purinergic signaling (Kamran et al., 2021). To this point, there remains substantial untapped therapeutic potential for P2XR modulation. Using the currently published structures as a roadmap, we have discussed the known targetable sites and postulated potential areas for SBDD targeting P2XRs. Continued structural investigation will advance our understanding of P2XR biology and reveal novel approaches for receptor modulation and therapeutic intervention.
